# Glycated hemoglobin level is significantly associated with the severity of coronary artery disease in non-diabetic adults

**DOI:** 10.1186/1476-511X-13-181

**Published:** 2014-12-04

**Authors:** Anping Cai, Guang Li, Jiyan Chen, Xida Li, Xuebiao Wei, Liwen Li, Yingling Zhou

**Affiliations:** Department of Cardiology, Guangdong Cardiovascular Institute, Guangdong General Hospital, Guangdong Academy of Medical Sciences, 106 Zhongshan Road 2, Guangzhou, 510080 China

**Keywords:** Glycated hemoglobin, Coronary artery disease, Diabetes mellitus

## Abstract

**Background:**

To investigate relationship between glycated hemoglobin (HbA1c) level and coronary artery disease (CAD) severity.

**Methods:**

Observational study was conducted and 573 participants were enrolled and baseline characteristics were collected. Clinical presentations in terms of stable angina, unstable angina or acute myocardial infarction were diagnosed. All participants were performed coronary angiography to figure out the numbers of coronary artery stenosis in terms of none-stenosis (< 50% stenosis), single or multiple vessels stenoses (≥ 50% stenosis). All participants were divided into subgroups according to two categories in terms of severity of clinical presentation (stable angina, unstable angina, or acute myocardial infarction) and the number of coronary artery stenosis (none, single, and multiple vessels). Primary endpoint was to evaluate relationship between baseline HbA1c value and CAD severity.

**Results:**

Consistent to previous studies, participants with CAD had more risk factors such as elderly, smoking, low HDL-C and high CRP levels. Notably, HbA1c level was more prominent in CAD group than that without CAD. As compared to stable angina subgroup, HbA1c levels were gradually increased in unstable angina and acute myocardial infarction groups. Similar trend was identified in another category in terms of higher HbA1c level corresponding to more vessels stenoses. Multivariate regression analyses showed that after adjusted for traditional risk factors as well as fasting blood glucose, HbA1c remained strongly associated with the severity of CAD. Nonetheless, there was no significant association when CRP was accounted for.

**Conclusion:**

HbA1c may be a useful indicator for CAD risk evaluation in non-diabetic adults.

## Introduction

Diabetes mellitus is a major and well established risk factor for macro-vascular diseases such as atherosclerotic cardiovascular diseases (ASCVD) and micro-vascular diseases such as neural, renal and retinal diseases [1, 2]. Of note, in patients with diabetes, plasma glucose level is significantly associated with the incidence and severity of micro-vascular and macro-vascular complications [1]. Currently, owing to its advantages over fasting blood glucose such as low intra-individual variability and being capable of evaluating the long-term blood glucose control [3, 4], glycated hemoglobin (HbA1c), a parameter of average blood glucose levels over 12 weeks, has also been suggested using in clinical practice currently [5, 6]. Evidence from epidemiological studies also showed that as compared to fasting blood glucose, HbA1c was more strongly associated with the risks of ASCVD and mortality from any causes [7, 8], which further supported the notion that HbA1c was superior to fasting blood glucose in predicting ASCVD outcomes. Nevertheless, some drawbacks regarding HbA1c, including un-satisfactory correlation with fasting blood glucose and over-diagnosis among patients with anemia and renal dysfunction, have also aroused concerns with respect to its application for diabetic diagnosis and ASCVD risk prediction [9, 10]. And the truly relationship between HbA1c and cardiovascular outcomes in populations without diabetes are still uncertain. For example, Silbernagel et al. reported that HbA1c significantly and independently of fasting blood glucose predicted all-cause of cardiovascular death in white population without diabetes [7]. Garg N and colleagues showed that in non-diabetics, HbA1c level has a linear incremental association with ASCVD [11]. Nonetheless, data from the Emerging Risk Factors Collaboration revealed that HbA1c merely added little incremental benefit for ASCVD risk prediction in patients without known ASCVD and diabetes [12].

In light of previous findings, our current study was designed to evaluate whether HbA1c level was associated with the severity of coronary artery diseases (CAD) in populations without diagnostic diabetes, and we believed that the clinical implication of our study would add, if any, valuable information to address whether HbA1c level could be used to predict the ASCVD risk in non-diabetic population.

## Methods

### Studied populations and protocols

Studied populations were enrolled from June of 2013 to April of 2014 after informed consent was obtained, and this study was approved by the Ethical Committee of Guangdong General Hospital. Current research is a retrospective and observational research, therefore, the including criteria are subjects with definitive coronary artery condition in terms of none-significant, single or multiple vessels stenoses as assessed by angiographic examination, and the excluding criteria are subjects without angiographic examination, with significant hepatic or renal dysfunction, anemia, tumor or connective tissue diseases. Clinical presentations in terms of stable angina, unstable angina or acute myocardial infarction were diagnosed according to their clinical manifestation, electrocardiography characteristics and cardiac biomarkers by two experts of cardiology. All participants were performed coronary angiography to definitely figure out the numbers of coronary artery stenosis in terms of none-significant (< 50% stenosis), single or multiple vessels stenoses (≥ 50% stenosis). Baseline characteristics including age, gender, family history of ASCVD, smoking status, hypertension, serum levels of lipid profile, fasting blood glucose, C-reactive protein (CRP) and HbA1c were collected at admission. In order to better understand whether HbA1c level was associated with the severity of CAD, all participants were divided into different subgroups according to two major categories in terms of the severity of clinical presentation (stable angina, unstable angina, or acute myocardial infarction) and the number of coronary artery stenosis (none, single, and multiple vessels stenoses).

### Studied endpoint

The primary endpoint of current study was to evaluate the relationship between HbA1c level and the severity of CAD. In addition, whether HbA1c was an independent risk indicator for the severity of CAD was also evaluated.

### Statistical analyses

Continuous data was presented as mean ± SD or median (interquartile range) appropriately, and compared by the Student’s t-test when data was normally distributed, otherwise compared by the Wilcoxon rank-sum test. Categorical data was presented as percentage and compared by χ^2^ test. Univariate and multivariate regression analyses were performed to evaluate the relationship between HbA1c value and the severity of coronary artery diseases. Statistical analyses were performed by using SPSS software version 16.0 (SPSS, Inc., Chicago, Illinois). A value of p < 0.05 was considered significant.

## Results

### Baseline characteristics

Totally 573 participants were enrolled and initially divided into two groups, namely without CAD group and CAD group, according to their coronary angiography examination. Since our current study was an observational study and therefore it was understandable and rational that the risk factors or comorbidities were more prevalent in the CAD group than that of without CAD group (as presented in Table 1). In the CAD group, age was older, male predominance, higher percentage of smoking and lower high density lipoprotein-cholesterol (HDL-C) level. No significant difference of total cholesterol (TC), triglyceride (TG), low density lipoprotein-cholesterol (LDL-C), renal function and fasting blood glucose were found between these two groups, except serum levels of CRP and HbA1c were significantly higher in the CAD group than that of without CAD group. Baseline characteristics among clinical subgroups were compared additionally. As presented in Table 2, risk factors including aging and smoking were more prevalent in the acute myocardial infarction group as compared to the stable angina group (p < 0.05). Serum level of CRP was significantly higher while apoA level was profoundly lower in the acute myocardial infarction when compared to the table angina group (p < 0.05).

**Table 1:** **Baseline characteristics**

Variable	Without CAD (n = 107)	CAD (n = 466)	P value
Age (years)	60.5 ± 12.1	63.83 ± 11.0	0.006
Male (%)	66 (61.7)	355 (76.2)	0.002
Family history (%)	5 (4.7)	32 (6.9)	0.405
Smoking (%)	27 (25.2)	173 (37.1)	0.020
Hypertension (%)	50 (46.7)	273 (58.6)	0.260
TG (mmol/L)	1.51 ± 1.05	1.78 ± 1.50	0.078
TC (mmol/L)	4.44 ± 1.01	4.30 ± 1.13	0.264
LDL-C (mmol/L)	2.67 ± 0.87	2.62 ± 0.99	0.609
HDL-C (mmol/L)	1.11 ± 0.30	0.97 ± 0.23	< 0.001
ApoA (mmol/L)	1.24 ± 0.39	1.10 ± 0.31	<0.001
ApoB (mmol/L)	0.81 ± 0.60	0.77 ± 0.23	0.273
CRP (mg/L)	3.67 ± 0.60	12.12 ± 1.21	0.001
Cr (umol/L)	96.5 ± 10.1	97.2 ± 8.8	0.228
BUN (mmol/L)	6.60 ± 1.33	6.82 ± 1.07	0.164
FBG (mmol/L)	5.58 ± 0.76	5.26 ± 0.69	0.178
HbA1c (%)	6.05 ± 1.17	6.55 ± 1.38	0.001

Denote: TG = triglyceride, TC = total cholesterol, LDL-C = low density lipoprotein-cholesterol, HDL-C = high density lipoprotein-cholesterol, Cr = creatinine, BUN = blood urine nitrogen.

**Table 2:** **Comparisons of baseline characteristics of clinical sub-groups**

Variable	None (n = 129)	Single (n = 87)	Multi (n = 250)	***P***value
Age (years)	59.7 ± 10.4	61.6 ± 11.3	64.2 ± 9.7	0.024
Male (%)	90 (69.8)	64 (73.6)	355 (80.4)	< 0.001
Family history (%)	8 (6.2)	6 (6.9)	32 (7.2)	0.113
Smoking (%)	41 (31.8)	33 (37.9)	99 (39.6)	0.036
Hypertension (%)	72 (55.8)	52 (59.8)	149 (59.6)	0.218
TG (mmol/L)	1.62 ± 0.21	1.71 ± 0.18	1.73 ± 0.22	0.133
TC (mmol/L)	4.65 ± 1.02	4.59 ± 1.01	4.70 ± 1.05	0.428
LDL-C (mmol/L)	2.82 ± 0.62	2.84 ± 0.55	2.86 ± 0.57	0.550
HDL-C (mmol/L)	1.08 ± 0.26	0.99 ± 0.24	0.95 ± 0.20	0.446
APOA (mmol/L)	1.29 ± 0.41	1.22 ± 0.36	1.13 ± 0.43	0.023
APOB (mmol/L)	0.82 ± 0.56	0.80 ± 0.35	0.80 ± 0.47	0.204
CRP (mg/L)	9.24 ± 1.08	10.86 ± 1.12	13.65 ± 1.17	< 0.001
Cr (umol/L)	96.8 ± 8.1	97.0 ± 6.4	97.6 ± 8.2	0.523
BUN (mmol/L)	6.67 ± 1.03	6.80 ± 1.01	6.82 ± 1.03	0.667
FBG (mmol/L)	5.22 ± 0.44	5.26 ± 0.38	5.29 ± 0.62	0.216

### Comparison of HbA1c levels among subgroups of CAD

**Figure 1:**
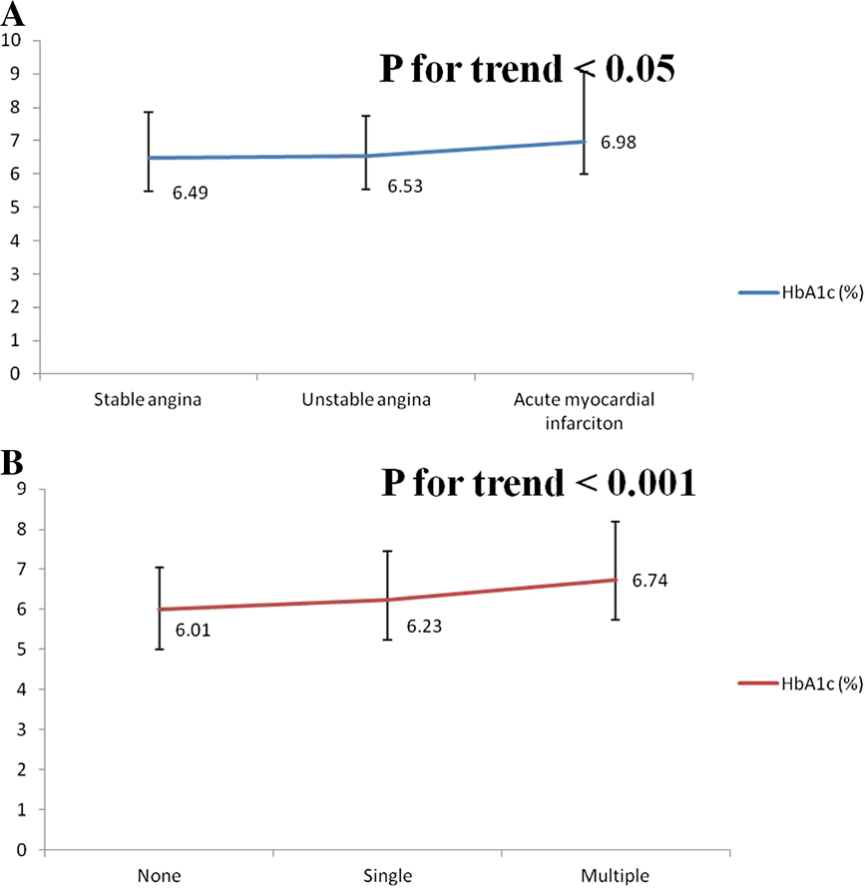
**Comparison of HbA1c levels among each subgroup in the two categories. Panel A**, HbA1c levels comparison among stable, unstable and AMI groups. **Panel B**, HbA1c levels comparison among none, single, and multiple vessels stenoses groups.

In order to evaluate the relationship of HbA1c level and severity of CAD, all CAD patients (n = 466) were divided into different subgroups according to two major categories as described above. In the first category, all CAD patients were divided into stable angina, unstable angina and acute myocardial infarction groups. As shown in Figure 1A, the difference of HbA1c level among each subgroup was significantly different, with a p-trend value < 0.05. In the second category, all CAD patients were divided into none-significant stenosis, single vessel stenosis and multiple vessels stenoses groups. As presented in Figure 1B, the HbA1c level was significantly associated with the number of coronary artery stenosis, with a p-trend value < 0.001.

### Multivariate regression analyses

For the purpose of investigating whether HbA1c was an independent risk factor for the severity of CAD, multivariate regression analyses were performed. In the first category, after adjustment for age, gender, family history of CAD, smoking and hypertension (in model 1a), HbA1c was strongly associated with the severity of CAD (P for trend = 0.010). Additional adjustment for TC, HDL-C, ApoA, and fasting blood glucose, the association was attenuated but HbA1c remained significantly associated with the severity of CAD (P for trend = 0.043, in model 1b). Notably, with adjustment for CRP (P for trend = 0.265, in model 1c), there was no significant association between HbA1c and the severity of CAD. In the second category, after adjustment for age, gender, family history of CAD, smoking and hypertension (in model 2a), there was a strongly relationship between HbA1c and the number of coronary artery stenosis (P for trend < 0.001). Additional adjustment for TC, HDL-C, ApoA, and fasting blood glucose (in model 2b), HbA1c remained strongly associated with the number of coronary artery stenosis (P for trend < 0.001). Similarly, with adjustment for CRP (P for trend = 0.168, in model 2c), there was no significant association between HbA1c and the number of coronary artery stenosis.

## Discussion

Currently, the predictive value of HbA1c on ASCVD risk in non-diabetic population is still less certain, and the application of HbA1c on ASCVD risk assessment among different Cardiovascular Society is also still inconsistent. Findings from our current study show that there is a significant association between HbA1c level and the severity of CAD in non-diabetic population, even after adjustment for traditional risk factors, and this relationship is independent of fasting blood glucose. Nonetheless, we also observed that after adjustment for CRP, there was no significant association between HbA1c and the severity of CAD.

Knowingly, glycated hemoglobin is a stable parameter reflecting approximately 3 months of blood glucose levels and it has now been suggested using in clinical practice for long-term blood glucose assessment in diabetic patients [1, 13]. HbA1c is calculated as the ratio of glycated to non-glycated N-terminal peptide of hemoglobin, and the HbA1c level has been universally recognized as important indicator for micro-vascular complications in diabetic patients [13, 14]. Nevertheless, whether HbA1c level could also be used in macro-vascular diseases (such as ASCVD) risk prediction is less clear, especially in non-diabetic populations [15, 16].

Accordingly, ASCVD is the leading cause of morbidity and mortality worldwide and improvement of risk discrimination is imperative [17]. Previously, results from previous studies showed that for coronary heart diseases, risk discrimination was significantly enhanced when HbA1c was added in risk algorithm in non-diabetic patients [8, 18, 19]. Our current study also showed that as compared to stable episode (stable angina), the levels of HbA1c were gradually and significantly increased in unstable conditions in terms of unstable angina and acute myocardial infarction, indicating that increased HbA1c level might result in atherosclerotic plaque pro-rupture, and to our best knowledge there were some mechanisms might partially explain this finding. Biologically, glycated hemoglobin is an advanced glycosylation end-product, and increased HbA1c level could reflect more generation of advanced glycosylation end-product, which might subsequently abundantly attached to vessel wall causing endothelial dysfunction and oxidative stress promotion [20, 21]. On the other hand, the binding of advanced glycosylation end-product might also result in inflammatory cytokines such as CRP over-production [22]. Increased CRP level has been found significantly associated with the instability of plaque [23, 24], and this might partially explain why after adjustment for CRP, there was no significant association between HbA1c and the severity of CAD. Finally, increased advanced glycosylation end-product could interfere with endogenous fibrinolytic system which might result in higher risk of coronary artery stenosis [25, 26]. Future experimental and clinical studies are warranted to investigate whether reduced HbA1c level will improve atherosclerosis plaque stability.

Other than the significant association between HbA1c level and the severity of CAD, our current study also showed that increased HbA1c level was associated with more vessel stenoses in non-diabetic patients. As aforementioned, by means of increasing vascular permeability, promoting endothelium dysfunction, and enhancing inflammation and oxidative reaction, increased advanced glycosylation end-product might lead to more vessels stenoses.

Consistent with previous some epidemiological studies [11, 18, 19, 27], data from our study also revealed that after adjusted for traditional risk factors including age, smoking, TC, HDL-C, HbA1c remained strongly associated with the severity of CAD. Notably, even after adjusted for fasting blood glucose, an independent risk factor for ASCVD and diabetes, there was still significant relationship between HbA1c and the severity of CAD, further supporting previous findings that HbA1c might be superior to fasting blood glucose in the respects of CVD risk discrimination. However, there was one distinctive discrepancy between our research and previous studies [11, 18, 19, 27] were that we concomitantly evaluated the relationship between HbA1c level and CAD severity including clinical scenario severity and the number of coronary artery stenosis.

As compared to fasting blood glucose, HbA1c level reflecting both fasting and post-prandial blood glucose might also partially explain the superiority of HbA1c, because peak plasma glucose level might cause more severe damage to endothelium [28, 29]. Future study is warranted to investigate whether there is significant interaction between post-prandial blood glucose and HbA1c on CVD risk prediction. Notably, there was no significant association between HbA1c and the severity of CAD when adjusted for CRP. The underlying mechanism might be ascribed to the increased CRP release when advanced glycosylated end-product was elevated.

## Conclusion

Our observational study found out that in non-diabetic population, HbA1c level was significantly associated with the severity of coronary artery diseases, which further supporting the notion that HbA1c may be a useful and independent indicator for CVD risk evaluation.
